# Effects of Gait Strategy and Speed on Regularity of Locomotion Assessed in Healthy Subjects Using a Multi-Sensor Method

**DOI:** 10.3390/s19030513

**Published:** 2019-01-26

**Authors:** Marco Rabuffetti, Giovanni Marco Scalera, Maurizio Ferrarin

**Affiliations:** IRCCS Fondazione Don Carlo Gnocchi, Milano 20121, Italy; gscalera@dongnocchi.it (G.M.S.); mferrarin@dongnocchi.it (M.F.)

**Keywords:** wearable/inertial sensors, accelerometer, regularity, variability, human, motion, locomotion, autocorrelation

## Abstract

The regularity of pseudo-periodic human movements, including locomotion, can be assessed by autocorrelation analysis of measurements using inertial sensors. Though sensors are generally placed on the trunk or pelvis, movement regularity can be assessed at any body location. Pathological factors are expected to reduce regularity either globally or on specific anatomical subparts. However, other non-pathological factors, including gait strategy (walking and running) and speed, modulate locomotion regularity, thus potentially confounding the identification of the pathological factor. The present study’s objectives were (1) to define a multi-sensor method based on the autocorrelation analysis of the acceleration module (norm of the acceleration vector) to quantify regularity; (2) to conduct an experimental study on healthy adult subjects to quantify the effect on movement regularity of gait strategy (walking and running at the same velocity), gait speed (four speeds, lower three for walking, upper two for running), and sensor location (on four different body parts). Twenty-five healthy adults participated and four triaxial accelerometers were located on the seventh cervical vertebra (C7), pelvis, wrist, and ankle. The results showed that increasing velocity was associated with increasing regularity only for walking, while no difference in regularity was observed between walking and running. Regularity was generally highest at C7 and ankle, and lowest at the wrist. These data confirm and complement previous literature on regularity assessed on the trunk, and will support future analyses on individuals or groups with specific pathologies affecting locomotor functions.

## 1. Introduction

Human functional periodic movements include locomotion, which is usually described in relation to its fundamental period [1], and upper limb activities, particularly those related to working tasks, sports, or art performances [2]. However, it is also possible to observe non-functional periodic movements such as tremor [3] or some cyclic gestures related to dystonic syndromes [4]. The periodicity of human locomotion is normally automatic and not necessarily consciously planned, controlled, and performed since specific neural structures, the central pattern generators, are in charge of controlling locomotor movements [5]. Moreover, inherent passive biomechanical and inertial characteristics of the different body parts naturally support the occurrence of passive pendulum-like periodic movements [6].

While, strictly speaking, a periodic phenomenon/signal repeats itself every predefined period of time *T*, a human cyclic movement and, particularly, kinematic and dynamic variables related to it are only approximately periodic; small variations occur both in the time domain and in the physical domain of the phenomena (*y*), according to

(1) y(t+T+ΔT)=y(t)+Δy,

where *y* is a generic gait analysis variable (with the exception of point trajectories, but including their derivatives), *T* is the fundamental period, and *t* is the time variable. Smaller values of *ΔT* and *Δy* indicate the movement (described by a variable y) being closer to a periodic phenomenon. Therefore, since *ΔT* and *Δy* only tend to zero, it is more proper to refer to the “pseudo-periodicity” of human movements [7]. In the scientific literature, such a unique aspect was assessed by different quantitative methods and related numerical indexes, concerning variability [8,9,10,11,12,13], stability [13,14], and regularity [12,15,16,17,18,19,20,21].

The variability of a pseudo-periodic movement may be increased due to endogenous physiological factors, like dual-task interference [13], or by pathological factors, such as those associated with neurodegenerative diseases [10]. Therefore, the quantification of the alteration of movement periodicity, i.e., the regularity of variables related to the performance, may represent a pathological marker to be considered in the clinical decision-making process [22], but only if confounding factors are taken into consideration.

Referring to gait, many studies addressed the variability of gait-related temporal parameters both in healthy individuals, to explore the possible influence of age [15] and different gait strategies (i.e., walking and running) [23], and in specific pathologic conditions, e.g., in frail elderly people [24], in people with neurological diseases, either degenerative [10] or focal [25], and in persons suffering from orthopedic diseases [12]. Interestingly, gait analysis methodologies based on wearable sensors [26] can efficaciously support quantitative assessment of variability and regularity; acceleration and angular velocity of anatomical parts, which are directly measured by inertial units, are characterized by pseudo-periodic patterns according to the pseudo-periodic nature of the considered locomotor act [27].

The study of locomotion regularity is generally based on an established time-domain approach involving autocorrelation analysis; movement regularity is assumed as the degree of similarity between two consecutive patterns of variables or signals characterizing the same cyclic movement [28,29]. Particularly, the measured acceleration undergoes an autocorrelation analysis [17,28] or an autocovariance analysis [29], whose outcome functions are characterized by a peak in correspondence of the fundamental period of the signal itself; the greater the peak Y-value is, the more regularly the signal pattern repeats. This latter number, thus, represents a regularity index. This method was successfully applied to the assessment of various samples of people, including elderly [19,30] and persons with locomotor disturbances [17,21,31,32,33]. 

Many factors, particularly gait speed [9,20,27] and gait strategy, i.e., walking or running [23], but also cognitive loads [20] and shoe type [16], were identified as modulating gait regularity. Moreover, when tracking regularity during long-term monitoring, an effect of fatigue on regularity was shown [34]. Furthermore, in some specific cases such as a musical performance, the regularity can be voluntarily modulated [2]. 

As to the sensor position, few studies considered more than one sensor placed on the trunk or pelvis to study head stabilization [35] or to assess across-sensor agreement [36]. All the cited articles about regularity reported analysis on single components of acceleration, thus requiring the identification of the meaningful acceleration components and the related accurate sensor alignment with the considered anatomical plane. Moreover, only sensors located on the trunk or pelvis were considered, and were, thus, unable to report regularity for limb movements.

The present study’s aims were as follows:

(A) To define a method to assess regularity by generalizing and further developing the already proposed method based on autocorrelation analysis [29]; innovative aspects include (1) the autocorrelation analysis being applied to the module of acceleration (i.e., norm of the acceleration vector) and not to one acceleration component, thus removing errors due to sensor misalignment; (2) a multi-sensor approach which enables comparatively studying the regularity of anatomical parts, particularly of upper and lower limbs; 

(B) To perform experiments and analyses on healthy subjects to quantify the effect of factors “strategy” (walking vs. running), “speed” (four speeds considered) [37], and “sensor location” (synchronized sensors located on pelvis, the seventh cervical vertebra (C7), and lower and upper limbs) on the regularity of human pseudo-periodic locomotor movements.

This study outcome may support future studies on human locomotion regularity by providing a robust methodology and reference data. Moreover, the application of the method to the study of non-functional periodic movements, such as tremors, is straightforward.

## 2. Materials and Methods

### 2.1. Measurement System

The measurement system used was the WaveTrack Inertial System (Cometa Systems, Italy; *https://www.cometasystems.com/*), which supported the four-sensor setting required for this study. The single device was a low weight (5.3 g), small-sized (32 mm × 24 mm × 7 mm), waterproof, inertial measurement unit (IMU; composed of a triaxial accelerometer, gyroscope, and magnetometer) with a wireless interface for real-time data streaming and a 1 GB of on-board memory, able to store up to 6 h of measurements. In the present study, only measurements of the triaxial accelerometer were considered; the sampling rate was 140 Hz with a 16-bit resolution analog-to-digital converter (ADC) and a full scale of ±16 g. Velcro elastic straps were provided for attaching sensors on selected anatomical landmarks.

### 2.2. Participants, Motor Tasks, and Raw Data

Twenty-five healthy subjects (14 males and 11 females, age range of 20–40 years) were recruited for the experiment (see details in Table 1). They signed an informed consent under the approval of the local Ethics Committee.

**Figure 1:**
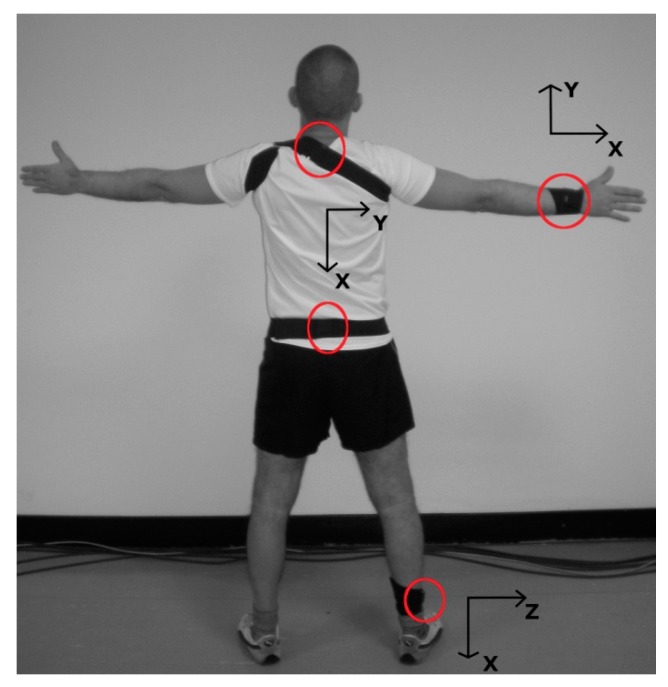
Subject equipped with four wearable triaxial accelerometers (Cometa Systems, Italy) positioned with elastic bands on the seventh cervical vertebra (C7), pelvis, wrist, and ankle. Sensor positions are circled in red, and axis directions are reported nearby.

Sensors were attached, by their flat surface, to the following landmarks: posterior aspect of spinous process of the seventh cervical vertebra (C7), midpoint between posterior superior iliac spines (pelvis), on dorsal aspect of the right wrist (wrist), and on lateral aspect of right ankle (ankle) (see Figure 1). Sensors were positioned in order to align their *X*-axes with longitudinal/vertical anatomical axes, sensor *Z*-axes were perpendicular to the sensor flat surface and were oriented accordingly.

The experiments were carried out on a treadmill and consisted of steady-state walking at 1.0, 1.4, and 1.8 m/s, and of steady-state running at 1.8 and 2.2 m/s (trials respectively labeled W1, W2, W3, R3, and R4). After taking between 30 and 60 s to reach steady-state locomotion, the duration of measurements was fixed at 70 s, the initial and final five seconds sections were discarded, and the further analysis was performed on the central one-minute-long recording. Trial sequences were randomly balanced among subjects; half of the participants performed the sequence W1, W2, W3, R3, and R4, and half performed the reversed sequence from R4 to W1.

Measured accelerometric components were low-pass filtered (fourth-order Butterworth filter, cutoff frequency 5 Hz, 0.5 dB of peak-to-peak ripple in pass-band, and 20 dB of attenuation in stop-band). Finally, acceleration module *a* was computed as the norm of the three measured orthogonal components (*a_x_, a_y_*, and *a_z_*) according to the following formula:

(2) $$a=\left|\vec{a}\right|=\sqrt{a_{x}^{2}+a_{y}^{2}+a_{z}^{2}}.$$

The experimental dataset consisted of 500 recordings obtained from 25 subjects, each performing five tasks, and equipped with four sensors.

### 2.3. Algorithm

**Figure 2:**
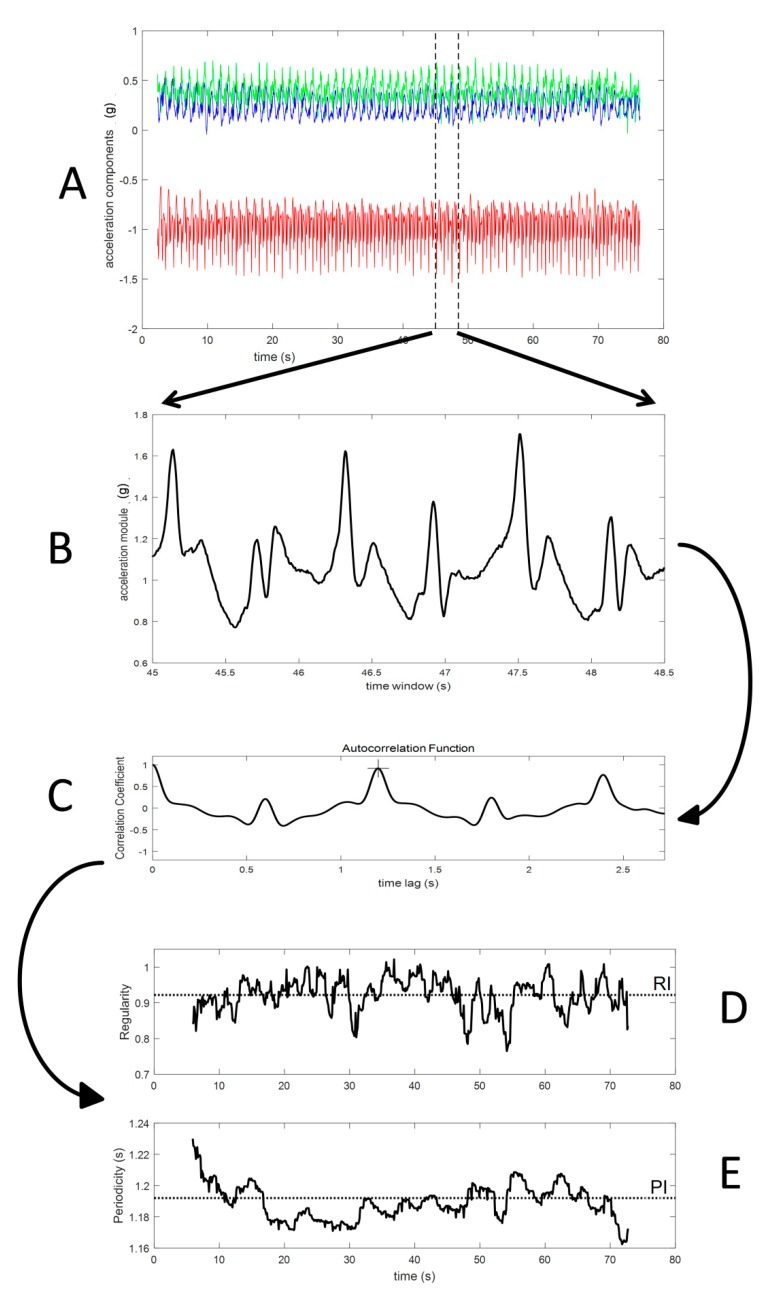
Method data flow: (**A**) raw recording from triaxial accelerometer; (**B**) window of acceleration module; (**C**) autocorrelation function computed on standardized windowed data (the cross marks the autocorrelation peak which corresponds to the window fundamental period); (**D**) the regularity time profile (the dotted line represents the window average value considered as a regularity index (RI)); (**E**) the fundamental period time profile (the dotted line represents the window average value considered as the period index (PI)).

The algorithm flowchart and sample data are presented in Figure 2. The input data consisted of the three acceleration components measured by a single sensor (Figure 2A). The analysis was applied on windowed finite time series of the acceleration module (Figure 2B) and of single acceleration components.

The duration (*n* seconds, or alternatively *N* samples) of the signal window was fixed, on each trial, to three times the fundamental period preliminarily quantified on the entire one-minute recording (this duration ranged from about 2 s for the fastest running to about 4 s for the slower walking). Such a choice was sufficient for a robust computation of the autocorrelation function and avoided regularity variations across a higher number of strides may have affected the index estimation [31].

The windowed data were first standardized (removed average and then divided by standard deviation) and then an autocorrelation analysis was applied, which is equivalent to an autocovariance analysis since a unitary covariance is obtained when correlating the signal with itself at zero lag. Thus, a unitary value of the autocorrelation function reflects a perfect regularity, while a null value marks the absence of any cyclic component, i.e., the absence of any regularity.

The autocorrelation sequence for a given data window (Figure 2C), which is a function of the sample lag *m*, was implemented as follows:

(3) $${\hat{R}}_{\mathrm{unbiased}}\left[m\right]=\frac{1}{N-m}\sum_{n=0}^{N-m-1}y\left[n+m\right]y\left[n\right],$$

where *R* is the autocorrelation function in the unbiased form, *y* is the analyzed variable, *N* is the number of data samples in the considered time window, and *m* is the number of samples quantifying the lag time of the autocorrelation function.

Notably, the previous equation includes a coefficient corrected for *m*, to compensate for the bias resulting from the *m* number of samples associated with the considered time lag (in fact, the larger the lag, the fewer samples support the computation) [29]. Negative lag values were not considered since they did not provide additional information, as autocorrelation is an even function.

Window periodicity, i.e., the fundamental period duration of the window data, corresponds to the time of the autocorrelation peak (Figure 2C). Window regularity corresponds to the peak value; the closer the input data are to a perfect periodic behavior, the closer the window regularity is to a unitary value. 

The previous analysis was then repeated for the subsequent data window which was time-shifted from the previous window by 0.1 s. This implies that two subsequent data windows, for locomotion recordings, may overlap for more than 95% of their data. In order to speed up and increase algorithm robustness, when considering the next data window, the previous identification of the autocorrelation peak was considered as the starting seed in the search of the actual peak of the autocorrelation function, thus substantially avoiding the identification of peaks related to higher harmonics of the fundamental period.

The regularity index (RI) and period index (PI) were computed as the average values of the sequences of window regularity (Figure 2D) and periodicity (Figure 2E), respectively.

All analyses were implemented in Matlab (version R2017b, The MathWorks Inc, USA).

### 2.4. Statistical Analysis

The outcome set included both the regularity index RI and the period index PI for all subjects, all tasks, and all sensor locations. As data were non-normally distributed, we used non-parametric tests; the Friedman test was used for non-parametric analysis of variance and the Wilcoxon test was used for post hoc and planned comparisons. The significance values (significance set to *p* ≤ 0.05) of all the tests inside the same analysis were corrected according to Holm–Bonferroni method. The experiment was designed to study the effect of three factors: a sensor factor, a speed factor, and a strategy (walking vs. running) factor. No analysis of interaction between factors was planned for two reasons: the sample data size was relatively small to allow efficaciously looking for interactions, and our interests were focused only on the main effects of the three factors.

As to the period index PI, a one-way non-parametric analysis of variance was performed to assess the across-sensor method robustness.

As to the regularity index RI, the experimental design allowed for assessing several factors. The analysis for the sensor factor was performed using one-way non-parametric analysis of variance. The speed factor was analyzed using the following planned comparison tests: W1 vs. W2, W2 vs. W3, and R3 vs. R4. Other comparisons were considered not relevant (W1 vs. W3) or inadequate (for example, W1 vs. R4) to explore the speed factor. The strategy factor (locomotion by walking vs. locomotion by running) was analyzed using a planned comparison of W3 vs. R3. Other comparisons (e.g., W2 vs. R4) report the effect of the strategy factor mixed with the effect of other factors (speed) and, therefore, were not included in the planned analysis.

Finally, in order to compare outcomes from the present module-based method with outcomes from a single-component approach in the regularity assessment, the algorithm was applied also to the single components of measured accelerations. Starting from RI_X, RI_Y, and RI_Z (regularity indexes of X, Y, and Z components, respectively), the minimum (worst index value) and the maximum (best index value) of the three single-component regularity indexes were identified for each subject/task/sensor, and multiple comparisons were performed between the module-based regularity index and the best and worst single-component ones.

## 3. Results

**Figure 3:**
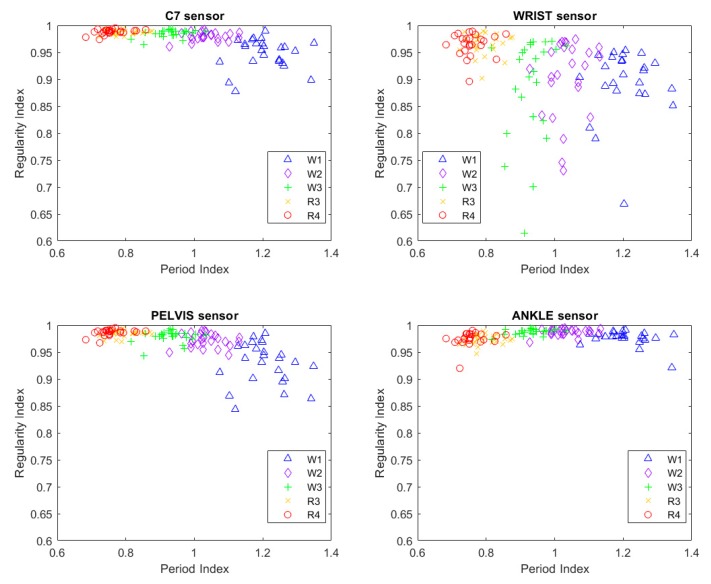
Summary scatterplot of all experimental outcomes: the period index is reported on the *X*-axis and the regularity index is reported on the *Y*-axis. Data from the four sensors are reported in separate plots. Different markers/color coding (refer to embedded legend) identify trial conditions (W1, W2, and W3 are for walking at 1.0, 1.4, and 1.8 m/s, respectively; R3 and R4 are for running at 1.8 and 2.2 m/s, respectively). The regularity index evidences an apparent ceiling effect.

A summary of the experimental data is presented in Figure 3; the scatterplot of the regularity index vs. period index clearly shows the clusters related to speed and strategy, while the data concerning the four different sensors are partially overlapped. The non-normal distributions of RI values are made apparent by the ceiling effect toward the unitary value.

### 3.1. Period Index

**Figure 4:**
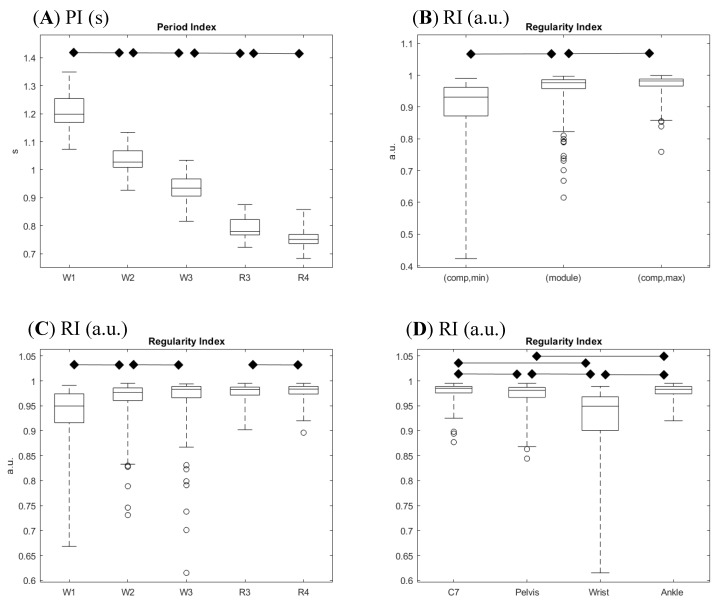
Composite presentation of results in 4 panels. (**A**) Period index (PI) distribution across tasks performed at different speeds and different modalities. (**B**) Comparison of the module-based regularity index (RI; center box) with the minimum (left box) and the maximum (right box) regularity indexes as computed on single components. (**C**) Regularity index distribution across tasks performed at different speeds and different modalities. (**D**) Regularity index distribution across sensor positions. Legends refer to tasks (W1, W2, and W3 for walking at 1.0, 1.4, and 1.8 m/s, respectively; R3 and R4 are for running at 1.8 and 2.2 m/s, respectively), to sensor positions (C7 on the posterior spinous process of the seventh cervical vertebra, pelvis on the midpoint between posterior superior iliac spine, wrist on the dorsal aspect of the right wrist, and ankle on the lateral aspect of right ankle), and to index definition (module for proposed computation on module of acceleration vector, min and max for extreme outcomes in single acceleration component computation). Boxes report medians and quartiles, whiskers represent extremes, and dots represent outliers. Horizontal bars with diamonds mark significant differences (*p* ≤ 0.05, corrected for multiple tests) according to planned statistical analysis.

The fundamental period estimation, quantified by the PI value, showed the expected modulation in relation to task modality and speed (Figure 4A); period decreased with increasing speed and, at a matched speed of 1.8 m/s, the period was shorter when running compared to walking (R3 vs. W3). The period estimation as obtained on the same trial from different sensors may provide different values; when considering the four values relative to the four different anatomical landmarks, the difference between the two extreme values was null in 46% of the trials, and accounted for 1 ms in 44% of the trials; only in 13 out of 125 trials was the across-sensor period estimate difference between 2 and 5 ms. A data perusal allowed identifying the wrist and ankle as the sensor locations responsible for larger (≥2 ms) deviations in period estimation.

### 3.2. Regularity Index: Comparison of Module-Based and Component-Based Analyses

Figure 4B reports a summary of the regularity index values obtained from the analysis of acceleration module pattern, along with the min and the max regularity indexes obtained from single acceleration components. A perusal of outcomes shows that the module-based regularity index was higher or equal to any component-based index computed on the same recording in about half of the trials. The statistical analysis evidenced an intermediate rank of the module-based index, though its median value was much closer to the best component index (−1.0%) compared to the worst component index (+9.5%). 

### 3.3. Regularity Index: Effect of Locomotion Speed

The effect of speed on regularity was highlighted by the comparisons between trials with the same locomotor strategy and with contiguous speeds (see Figure 4C). The tested couples were W1 vs. W2, W2 vs. W3, and R3 vs. R4. Regularity during walking trials significantly increased with increasing velocity, getting very close to the unit value for higher speeds. On the contrary, increasing velocity in running did not increase regularity. Nonetheless, it has to be noted that all values related to running showed a tendency to a ceiling effect with regularity indexes, limited in their values by the unitary upper limit, which marks a perfect regularity.

### 3.4. Regularity Index: Effect of Locomotor Strategy

The only test able to explore the effect of a different locomotor strategy was the comparison between the regularity indexes of W3 and R3, i.e., walking and running at the same velocity (1.8 m/s). This was the only feasible comparison, since healthy subjects can switch between walking and running without forcing themselves into a narrow range of speeds. No significant differences emerged from the Wilcoxon test, though a perusal of data distributions showed occasionally lower regularity in walking trials (see Figure 4C).

### 3.5. Regularity Index: Effect of Sensor Location

The statistical analysis showed a significant difference of regularity among locations; the highest regularity was found for C7 (median value 0.985) and the ankle (0.983), while the lowest was found for the wrist (0.949), and intermediate regularity was found for the pelvis (0.981) (Figure 4D). When further exploring the previous analysis by comparing the two locomotor strategies at a matched velocity, the median value of the regularity index increased in the running trials compared to the walking trials, at C7 (+1.0%), the pelvis (+1.6%), and the wrist (+4.8%), while it decreased (−1.0%) at the ankle.

## 4. Discussion

The present method for quantifying regularity in pseudo-periodic human movements was a direct evolution and a generalization of methods based on the autocorrelation analysis to study trunk oscillatory movements during locomotion [28,29]. The proposed method’s novelty is in the application of the autocorrelation analysis to the module of acceleration and to the concurrent measurement of acceleration on four different anatomical points.

The autocorrelation analysis was applied to the module of acceleration, derived from measurements by a triaxial sensor, which was periodic to the extent that single components are periodic. This novel aspect was expected to imply a larger robustness than single-component methods since the latter require an accurate placement for sensor orientation and location (sensor axes have to be aligned with anatomical or functional axes, and this may be particularly prone to operator errors in experiments involving persons with abnormal anatomical features). On the contrary, the acceleration module is independent from sensor orientation, but relies only upon sensor location. As to the computational aspects, the autocorrelation was applied to a time-finite windowed signal after having standardized it (subtracted average and divided by standard deviation), implying a unitary value for RI indicating perfect regularity. The average removal (equivalent to the autocovariance approach explicitly adopted by Moe-Nilssen [29]) is relevant, since an offset does corrupt, proportionally to the offset relevance/amplitude, the resulting autocorrelation coefficient and, thus, the regularity index. Therefore, this approach increases the robustness of the method.

The proposed sensor positions were not limited to location on the sagittal symmetry plane of the trunk–pelvis as in previous studies, but other body locations, particularly on either upper and lower limbs, were considered. This was possible since a periodic movement implies periodic patterns of the acceleration measured on any moving body location. A multi-sensor arrangement as already considered by Rispens et al. [36]; however, they only compared a regularity assessment of different locations on the trunk–pelvis segment, omitting the limbs. Obviously, locations outside the body’s sagittal symmetry plane cannot support the analysis of symmetry according to Reference [29]; however, the multi-point approach here proposed has the advantage of disclosing how different body parts contribute to the movement regularity, thus supporting understanding and potentially targeting treatment or training on specific body segments.

The proposed autocorrelation analysis produced an autocorrelation time profile (see Figure 1E) whose average on a one-minute-long track was considered here as a regularity index (RI). However, analysis on longer recordings produced longer regularity profiles, which could be further studied for the occurrence of trends, potentially unveiling effects of fatigue on regularity. Schutte already dealt with this aspect by simply analyzing two spot assessments before and after a prolonged fatiguing task [34]. Moreover, the multi-sensor approach may further support a fatigue analysis allowing the identification of which anatomical functional part acts as a fatigue trigger.

The experimental data were obtained from one-minute-long locomotion trials at constant velocity on a treadmill, whereas previous studies considered recordings of walking for one minute or less [17,28,29]. The subjects were randomly requested to perform the sequence from slower walking to faster running or the reverse-order sequence, and no bias resulted in regularity index. 

The experimental dataset also allowed, for all three components of acceleration singularly considered, to compute stride regularity according to the previous method [29]. For the sake of simplicity, the highest and the lowest outcome indexes as computed from single components were identified and then compared with the regularity indexes computed on the acceleration module pattern. The results show that the average difference between the highest single-component-based index and the module-based index was very little for the considered dataset, about 1% in relative terms, though statistically significant. However, no substantial bias was observed, since about half of the trials displayed a higher regularity index for the module-based index. Nonetheless, the option for a single-component regularity analysis, which showed validity in healthy subjects and in the study of selected locomotor disturbances [17,21,29], requires that this component be identified before the experiment takes place, which is a decision that may suffer from errors particularly when motor disturbances affect the performing subject. Ultimately, the proposed module-based analysis on a specific anatomical location [38] does not require that the researcher accurately align the sensor axes along predefined directions, which is particularly difficult when skeletal deformities affect the performing subject. Obviously, the adoption of triaxial accelerometers does not exclude the possibility to apply both the module-based approach and the single-component one.

Interesting findings emerged also from the analysis of period duration, a useful secondary outcome of the autocorrelation method. Firstly, it was confirmed that the estimate was very robust across sensors and across methods in accordance with published studies [39], and results were comparable with published normative data [40]. Secondly, the method provided a tracking of the fundamental period value along with the monitoring time; such information is supportive of studies concerning the gait variability [10]. Moreover, since the sensors may be placed on most parts of the body and adopted to any pseudo-periodic movements, this method appears to be of general applicability, while gait-specific methods for gait event identification cannot be extended to other motor tasks. 

The application of the method was intended to disclose if different body segments showed different regularity during locomotion, and to analyze if locomotion strategy and locomotion speed influenced movement regularity.

Since the experiments involved the synchronized measurement of acceleration from four sensors, we were able to compare the autocorrelation coefficients observed on different anatomical sites; the highest regularity was found for C7 and the ankle, while the lowest (but still very high in absolute terms) was found for the wrist. The interpretation of C7’s higher regularity may be related to biomechanical aspects (segments with larger mass and inertia, such as the trunk, show more regular movements, in the same manner that flywheels are expected to), to motor-control specific features (C7 movements are more regular because one of the objectives of locomotion is to keep the head, and the sensory organs inside it, the most stable and regular [35]) and to the balancing role played by limbs (particularly upper limbs) in keeping the body stability [41]. The reason for the lower regularity of upper limbs may include the possibility of accomplishing other episodic motor tasks, asynchronous with walking [42,43].

The effect of gait speed was assessed by comparing trials at different speeds but with the same strategy; a speed effect was observed for walking trials only, thus confirming that increasing speed is associated with increased regularity [20]. However, we observed that a faster locomotion resulted in the regularity index being closer to its maximum possible unitary value; therefore, it is reasonable to hypothesize the emergence of a ceiling effect at faster gait speeds, which could explain the missing detection of a speed effect for the running trials.

Interestingly, when comparing walking and running trials, the trunk (i.e., C7 and the pelvis) and, even more so, the upper arms tended to be more regular in running, according to the speed effect already evidenced, while lower limbs (ankle) were less regular. Such a decrease in regularity in lower limbs may be due to a technical artefact; the feet contacts on the ground are characterized, during running, by large impulsive impact forces which may transmit irregular oscillation on the nearby musculoskeletal structures and, therefore, also on the accelerometer tighten to the ankle [44].

As to the motor strategy, no between-strategy difference in regularity was observed at one single matched velocity close to the transition speed (RI_W3_ = RI_R3_ in Figure 4C), thus confirming previous studies concerning the analysis of spatio-temporal parameters [23]. While walking and running performances showed values of regularity index very close each other and close to the unitary upper limit value, a perusal of the whole dataset showed how RI never got below 0.9 when running, while 10 out of 100 RI values when walking were below 0.9, ranging down to approximately 0.7. Interestingly, all those ten values referred to regularity assessed by the wrist sensor. A possible explanation is that the role of upper limbs during running is more strictly related to locomotion than during walking, since they must counterbalance larger inertial forces which occur in the body [45]. Conversely, it can be expected that, during walking, the upper limbs keep the possibility to also perform some locomotor-unrelated tasks, characterized by less regular features [43].

## 5. Conclusions

The proposed method was proven to be feasible and reliable, to be able to quantify the regularity of movement of different anatomical parts, and to be able to track modulation of regularity determined by locomotor strategy and speed. The novel methodological approach of considering the acceleration module has the advantage over single-component methods [17,29] to be unaffected by sensor misalignment. The data from a group of healthy individuals may foster the collection of a larger dataset and provide reference in studies concerning pathological conditions.

Already planned future applications of the method include (a) a real-time application in providing biofeedback to the patient during periodic movements to help them in keeping a regular motor pattern (such as gait, but also upper limb movements), thus supporting motor learning rehabilitation protocols [46,47]; (b) studies on the modulation of regularity during prolonged performances, potentially induced by fatigue or by a voluntary change in motor strategy [48]; (c) studies on non functional pseudo-periodic movements, such as tremor or dyskinesia, by means of regularity analysis of long-term actigraphic recordings [49].

## Figures and Tables

**Table 1:** Participants’ demographic and anthropometric data. M—male; F—female.

Sex	Age (years)	Body Height (m)	Body Weight (kg)
M	31	1.62	52
M	26	1.76	85
F	22	1.61	45
F	29	1.60	49
M	32	1.88	86
M	40	1.62	48
F	27	1.67	53
M	30	1.70	61
M	24	1.81	75
F	28	1.85	73
M	35	1.75	75
M	26	1.70	57
F	20	1.74	59
F	23	1.79	65
M	24	1.70	70
M	25	1.89	83
M	26	1.80	74
M	25	1.70	75
F	22	1.62	46
M	29	1.74	75
F	26	1.61	45
F	23	1.60	51
F	23	1.60	55
M	24	1.80	79
F	23	1.70	55

## References

[1] Bovi G., Rabuffetti M., Mazzoleni P., Ferrarin M. (2011). A multiple-task gait analysis approach: Kinematic, kinetic and EMG reference data for healthy young and adult subjects. Gait Posture.

[2] Demos A.P., Lisboa T., Chaffin R. (2016). Flexibility of Expressive Timing in Repeated Musical Performances. Front. Psychol..

[3] Deuschl G., Bain P., Brin M. (1998). Consensus statement of the Movement Disorder Society on Tremor. Ad Hoc Scientific Committee. Mov. Disord..

[4] Albanese A., Bhatia K., Bressman S.B., Delong M.R., Fahn S., Fung V.S.C., Hallett M., Jankovic J., Jinnah H.A., Klein C. (2013). Phenomenology and classification of dystonia: A consensus update. Mov. Disord..

[5] Crenna P., Carpinella I., Lopiano L., Marzegan A., Rabuffetti M., Rizzone M., Lanotte M., Ferrarin M. (2008). Influence of basal ganglia on upper limb locomotor synergies. Evidence from deep brain stimulation and L-DOPA treatment in Parkinson’s disease. Brain.

[6] Usherwood J.R., Channon A.J., Myatt J.P., Rankin J.W., Hubel T.Y. (2012). The human foot and heel-sole-toe walking strategy: A mechanism enabling an inverted pendular gait with low isometric muscle force?. J. R. Soc. Interface.

[7] Pecoraro F., Mazzà C., Zok M., Cappozzo A. (2006). Assessment of level-walking aperiodicity. J. Neuroeng. Rehabil..

[8] Hausdorff J.M. (2005). Gait variability: Methods, modeling and meaning. J. Neuroeng. Rehabil..

[9] Jordan K., Challis J.H., Newell K.M. (2007). Walking speed influences on gait cycle variability. Gait Posture.

[10] Hausdorff J.M. (2009). Gait dynamics in Parkinson’s disease: Common and distinct behavior among stride length, gait variability, and fractal-like scaling. Chaos.

[11] Stergiou N., Decker L.M. (2011). Human movement variability, nonlinear dynamics, and pathology: Is there a connection?. Hum. Mov. Sci..

[12] Parker K., Hanada E., Adderson J. (2013). Gait variability and regularity of people with transtibial amputations. Gait Posture.

[13] Magnani R.M., Lehnen G.C., Rodrigues F.B., de Sá E Souza G.S., de Oliveira Andrade A., Vieira M.F. (2017). Local dynamic stability and gait variability during attentional tasks in young adults. Gait Posture.

[14] Bisi M.C., Riva F., Stagni R. (2014). Measures of gait stability: Performance on adults and toddlers at the beginning of independent walking. J. Neuroeng. Rehabil..

[15] Kobsar D., Olson C., Paranjape R., Hadjistavropoulos T., Barden J.M. (2014). Evaluation of age-related differences in the stride-to-stride fluctuations, regularity and symmetry of gait using a waist-mounted tri-axial accelerometer. Gait Posture.

[16] Terrier P., Dériaz O., Meichtry A., Luthi F. (2009). Prescription footwear for severe injuries of foot and ankle: Effect on regularity and symmetry of the gait assessed by trunk accelerometry. Gait Posture.

[17] Tura A., Raggi M., Rocchi L., Cutti A.G., Chiari L. (2010). Gait symmetry and regularity in transfemoral amputees assessed by trunk accelerations. J. Neuroeng. Rehabil..

[18] Kurz M.J., Hou J.G. (2010). Levodopa influences the regularity of the ankle joint kinematics in individuals with Parkinson’s disease. J. Comput. Neurosci..

[19] Kobayashi H., Kakihana W., Kimura T. (2014). Combined effects of age and gender on gait symmetry and regularity assessed by autocorrelation of trunk acceleration. J. Neuroeng. Rehabil..

[20] Schaefer S., Jagenow D., Verrel J., Lindenberger U. (2015). The influence of cognitive load and walking speed on gait regularity in children and young adults. Gait Posture.

[21] Barden J.M., Clermont C.A., Kobsar D., Beauchet O. (2016). Accelerometer-Based Step Regularity Is Lower in Older Adults with Bilateral Knee Osteoarthritis. Front. Hum. Neurosci..

[22] Cuzzolin F., Sapienza M., Esser P., Saha S., Franssen M.M., Collett J., Dawes H. (2017). Metric learning for Parkinsonian identification from IMU gait measurements. Gait Posture.

[23] Dingwell J.B., Bohnsack-McLagan N.K., Cusumano J.P. (2018). Humans control stride-to-stride stepping movements differently for walking and running, independent of speed. J. Biomech..

[24] Mortaza N., Abu Osman N.A., Mehdikhani N. (2014). Are the spatio-temporal parameters of gait capable of distinguishing a faller from a non-faller elderly?. Eur. J. Phys. Rehabil. Med..

[25] Sánchez M.C., Bussmann J., Janssen W., Horemans H., Chastin S., Heijenbrok M., Stam H. (2015). Accelerometric assessment of different dimensions of natural walking during the first year after stroke: Recovery of amount, distribution, quality and speed of walking. J. Rehabil. Med..

[26] Muro-de-la-Herran A., Garcia-Zapirain B., Mendez-Zorrilla A. (2014). Gait Analysis Methods: An Overview of Wearable and Non-Wearable Systems, Highlighting Clinical Applications. Sensors.

[27] Kim Y.-K., Joo J.-Y., Jeong S.-H., Jeon J.-H., Jung D.-Y. (2016). Effects of walking speed and age on the directional stride regularity and gait variability in treadmill walking. J. Mech. Sci. Technol..

[28] Auvinet B., Berrut G., Touzard C., Moutel L., Collet N., Chaleil D., Barrey E. (2002). Reference data for normal subjects obtained with an accelerometric device. Gait Posture.

[29] Moe-Nilssen R., Helbostad J.L. (2004). Estimation of gait cycle characteristics by trunk accelerometry. J. Biomech..

[30] Gillain S., Boutaayamou M., Dardenne N., Schwartz C., Demonceau M., Gerontitis C., Depierreux F., Salmon E., Garraux G., Bruyère O. (2017). Data set of healthy old people assessed for three walking conditions using accelerometric and opto-electronic methods. Aging Clin. Exp. Res..

[31] Tura A., Rocchi L., Raggi M., Cutti A.G., Chiari L. (2012). Recommended number of strides for automatic assessment of gait symmetry and regularity in above-knee amputees by means of accelerometry and autocorrelation analysis. J. Neuroeng. Rehabil..

[32] Yang C.-C., Hsu Y.-L., Shih K.-S., Lu J.-M. (2011). Real-time gait cycle parameter recognition using a wearable accelerometry system. Sensors.

[33] Demonceau M., Donneau A.-F., Croisier J.-L., Skawiniak E., Boutaayamou M., Maquet D., Garraux G. (2015). Contribution of a Trunk Accelerometer System to the Characterization of Gait in Patients with Mild-to-Moderate Parkinson’s Disease. IEEE J. Biomed. Health Inf..

[34] Schütte K.H., Maas E.A., Exadaktylos V., Berckmans D., Venter R.E., Vanwanseele B. (2015). Wireless Tri-Axial Trunk Accelerometry Detects Deviations in Dynamic Center of Mass Motion Due to Running-Induced Fatigue. PLoS ONE.

[35] Menz H.B., Lord S.R., Fitzpatrick R.C. (2003). Acceleration patterns of the head and pelvis when walking on level and irregular surfaces. Gait Posture.

[36] Rispens S.M., Pijnappels M., van Schooten K.S., Beek P.J., Daffertshofer A., van Dieën J.H. (2014). Consistency of gait characteristics as determined from acceleration data collected at different trunk locations. Gait Posture.

[37] Jin L., Hahn M.E. (2018). Modulation of lower extremity joint stiffness, work and power at different walking and running speeds. Hum. Mov. Sci..

[38] Della Croce U., Leardini A., Chiari L., Cappozzo A. (2005). Human movement analysis using stereophotogrammetry. Part 4: Assessment of anatomical landmark misplacement and its effects on joint kinematics. Gait Posture.

[39] Fortune E., Lugade V., Morrow M., Kaufman K. (2014). Validity of using tri-axial accelerometers to measure human movement—Part II: Step counts at a wide range of gait velocities. Med. Eng. Phys..

[40] Hansen E.A., Kristensen L.A.R., Nielsen A.M., Voigt M., Madeleine P. (2017). The role of stride frequency for walk-to-run transition in humans. Sci. Rep..

[41] Wu Y., Li Y., Liu A.-M., Xiao F., Wang Y.-Z., Hu F., Chen J.-L., Dai K.-R., Gu D.-Y. (2016). Effect of active arm swing to local dynamic stability during walking. Hum. Mov. Sci..

[42] Carpinella I., Crenna P., Rabuffetti M., Ferrarin M. (2010). Coordination between upper- and lower-limb movements is different during overground and treadmill walking. Eur. J. Appl. Physiol..

[43] Lamberg E.M., Muratori L.M. (2012). Cell phones change the way we walk. Gait Posture.

[44] Lafortune M.A. (1991). Three-dimensional acceleration of the tibia during walking and running. J. Biomech..

[45] Arellano C.J., Kram R. (2014). The metabolic cost of human running: Is swinging the arms worth it?. J. Exp. Biol..

[46] Scalera G.M., Rabuffetti M., Marzegan A., Frigo C., Ferrarin M. Regularity assessment of cyclic human movements: An innovative method based on wearable sensors. Proceedings of the 2017 E-Health and Bioengineering Conference (EHB).

[47] Jonsdottir J., Cattaneo D., Recalcati M., Regola A., Rabuffetti M., Ferrarin M., Casiraghi A. (2010). Task-oriented biofeedback to improve gait in individuals with chronic stroke: Motor learning approach. Neurorehabil. Neural Repair.

[48] Erdmann W.S., Lipinska P. (2013). Kinematics of marathon running tactics. Hum. Mov. Sci..

[49] Rabuffetti M., Meriggi P., Pagliari C., Bartolomeo P., Ferrarin M. (2016). Differential actigraphy for monitoring asymmetry in upper limb motor activities. Physiol. Meas..

